# Reimagining entryways: How work-based learning at Clinical and Translational Science Award (CTSA) centers is expanding access for the next generation of clinical research professionals

**DOI:** 10.1017/cts.2026.10806

**Published:** 2026-07-27

**Authors:** Kate Marusina, Olga Kishchenko, Michelle Jenkerson, Rachel Cody, Jessica Cranfill, Stephanie Freel, Shirley L.T. Helm, Fanny Delebecque, Anthony Keyes, Veronica Flock, Clare Tyson, Natalie Naylon, Erika J. Stevens

**Affiliations:** 1 Clinical and Translational Science Center, UC Davis, Davis, CA, USA; 2 Institute of Clinical and Translational Sciences, Washington University in St Louis, St Louis, MO, USA; 3 Duke Clinical and Translational Science Institute, Duke University School of Medicine, Durham, NC, USA; 4 C. Kenneth and Dianne Wright Center for Clinical and Translational Research, Virginia Commonwealth University, Richmond, VA, USA; 5 Altman Clinical and Translational Research Institute, University of California San Diego, La Jolla, CA, USA; 6 Institute for Clinical and Translational Research, Johns Hopkins University, Baltimore, MD, USA; 7 Office of Research Education, Mayo Clinic, Rochester, MN, USA; 8 South Carolina Clinical & Translational Research (SCTR) Institute, Medical University of South Carolina, Charleston, SC, USA; 9 New Jersey Alliance for Clinical Translation Science, Rutgers Health, Newark, NJ, USA

**Keywords:** clinical research, workforce development, training, model, internship, apprenticeship

## Abstract

The 2025 report by the Association for Clinical Research Professionals summarizes the state of crisis for the global clinical research workforce and proposes key solutions. These include increased awareness of clinical research professions, access to jobs through expanded training and internships, and advancement opportunities for professional staff and site support. To increase access to these professions, multiple Academic Medical Centers (AMCs) expanded training programs and internships leveraging approaches described in this manuscript, and in some cases, established formal apprenticeships in their respective States. This manuscript summarizes direct experiences of nine Clinical and Translational Science Award Centers for decreasing the barriers to entry into the clinical research profession, specifically focusing on clinical research coordinator role. This special communication describes methods for increased awareness, establishing training/internship opportunities, while addressing internal and external collaborations, administrative barriers, and funding necessary to implement an innovative pathway program. The authors hope that this material will be valuable to other AMCs that wish to undertake development of similar programs.

## Introduction

Clinical research professionals (CRPs) play a key role in the conduct of clinical research. This profession demands comprehensive knowledge, competencies, and skills to navigate increasing therapeutic, regulatory, and logistical complexities [1]. An Association for Clinical Research Professionals (ACRP) white paper calls for collective action to replenish the CRP workforce and establish academic and on-the-job training to meet escalating demands [2]. The national need for CRPs increases over 8% annually, far surpassing new entries into the workforce. This white paper specifically calls out CRP career access and advancement as critical imperatives. A key first step is to increase awareness given these biomedical research career paths are largely unknown to the public [2]. Community outreach and partnership building is an important strategy for launching a successful workforce pathway program. Formal or informal relationships with local Community-Based Organizations (CBOs), community colleges, and institutions of higher learning are essential for sparking interest in trainees/students. Often, such relationships support economic advancement for local communities [3].

Challenges at Academic Medical Centers (AMCs) may include authorizing the presence of students on the premises, administrative onboarding, and provision of stipends. Training challenges emerge in identifying and preparing trainers, attracting subject matter experts, creating appropriate training materials and gaining access to software environments. Thus, CRP pathway programs can quickly become a burden to hosting AMCs. The internationally-recognized Joint Task Force (JTF) Competency Domains present an opportunity to systematize development and share training curricula, whether developed in-house tailored to local policies and procedures, shared, or acquired via license [4].

Trainee’s needs must also be considered. Challenges for delivering informationally dense content in a short span of time include: a) preconceived notions about research, the academic environment, and dominance of cultural concepts; b) lack of higher education; c) and lack of knowledge regarding medical and scientific terminology. The required skillset for CRPs is gained through experience as academic curriculum alone may be insufficient to prepare trainees for entry-level positions [5]. Numerous Clinical and Translational Science Awards (CTSAs) utilize shadowing days, mock activities, or on-the-job training. Harmonizing the needs of study teams with the needs of trainees can be complicated, requiring additional checklists, written training goals, and frequent check-ins with research teams. A mentor can be a valuable link between students and programs. While completion of pathway programs frequently leads to interview opportunities, applicants may benefit from assistance with resume writing and interview preparation. Gathering meaningful metrics informs program success, areas for improvement, and long-term outcomes.

CRP pathway programs safeguard current and future staffing levels of the workforce. To support these programs, significant investment in funding, effort, and advocacy is needed from university leadership, CTSA faculty, and staff. The authors of this manuscript share their experiences in creating successful CRP pathway programs across unique AMC environments. We encourage other AMCs to consider these lessons learned in developing their own programs toward expanding a qualified and skilled CRP workforce. To our knowledge, this is the first manuscript that summarizes such collective experience.

## Materials and methods

In 2024, the Association of Clinical and Translational Science (ACTS), Clinical Research Professional Taskforce (CRPT) convened a working group to identify current practices for workforce development across CTSA institutions. Nine CTSA institutions with CRP pathway programs participated in monthly meetings to explore current practices. Group discussions provided insights on institutional capabilities, available infrastructure, and program design. Descriptive information and supporting documents were collated and analyzed for structural similarities as well as innovative and impactful approaches. The workgroup shared information across topic verticals: community engagement/ partnerships, administrative processes, training/ curriculum, delivery modality, program evaluation, and funding. Shared insights across these CTSA organizations frame the focus of this special communication. The workgroup agrees that a successful CRP pathway program consists of the following components (not in order of priority):

1. Community engagement and collaboration, including formalized partnerships
2. Administrative processes, including applicant selection, onboarding, and providing access to systems and technologies
3. Training curriculum, training staff, SMEs, and mentors
4. Shadowing, hands-on, and internship opportunities
5. Program evaluation
6. Funding

Below are the aggregated observed practices and examples for each of the components from author institutions. A tabular compilation of strategies for each component is available in Supplementary Materials and referenced in each Exemplar. The authors acknowledge that the majority of the described pathway program focus on training Clinical Research Coordinators (CRC). However, many principles apply for development of other professions under the CRP umbrella.

### Community engagement

Success of a workforce development program is highly dependent on identifying and engaging various collaborators. Whether the goal is to increase awareness of the program, apply for joint funding, or seek potential applicants; positive, well-structured partnerships provide vital connections with the local community. In some cases, CTSAs sought formal agreements to identify bidirectional expectations, define clear roles, responsibilities, and timelines, discuss release of liabilities, confidentiality provisions, access to medical records etc. Whether formal or not, any collaboration would benefit from outlining the goals and objectives, and their alignment with the overall CTSA Workforce Development goals. When collaborating with the CBOs, the goals are often focused on creating a pathway to employment as entry-level CRPs and increasing the representation of individuals from historically underrepresented backgrounds. Conversely, collaborations with academic entities may focus on introducing students to clinical research concepts and providing an overview of professional pathways in clinical research. Continuous reiteration of goals and objectives, and current responsibilities should be a part of the communication strategy. Establishment of recruitment criteria often needs to take into consideration the stipulation of a funding sponsor. For example, many local community development grants have age or residency restrictions. The duration and complexity of the training program would add additional selection pressure. Including stakeholders during interviews may reduce biases in student selection (Supplemental materials: tabular compilation of strategies).

#### Exemplar. Duke University: Building pathways through strategic educational partnerships (strategies CE7 and CE11, supplemental materials)

*The Duke Clinical and Translational Science Institute partners with North Carolina Central University and Durham Technical Community College (Durham Tech) to offer competency-aligned internships and apprenticeships [6]. Recently, these partnerships have founded a novel pathway program integrating into the Durham public school system to offer a health care and research focused school with direct-to-job opportunities upon graduation. It is the first early college in the region to include a clinical research pathway, offering students tuition-free access to both a high school diploma and college credit or credentials.*

#### Exemplar. UC Davis collaboration with community-based organizations reaches to communities underrepresented in research

*UC Davis established collaborations with two local CBOs (Strategy CE8, Supplemental Materials) to access potential candidates for the Join the Team training program [7]. Collaborations with the CBOs achieve multiple objectives, including alignment with the Anchor Institution mission of the UC Davis to train and hire locally. In addition, a CBO can provide eligibility verification, assist in completing necessary onboarding requirements, such as immunization and background check, and provide trainees with wrap-around services based on need (transportation, childcare, computers, and resume preparation). Close relationships with CBOs resulted in increased chances for trainees to complete the program.*

### Administrative processes, including applicant selection, onboarding, and providing access to systems and technologies

Applicant selection typically consists of an application and interview process and is often dictated by the funding mechanism. Involving collaborating organizations in this process is a valuable practice. While this may be fairly straightforward, in some cases students attending training programs are not affiliated with the University (i.e. not an undergraduate student). “Formalizing” such community student presence on medical campuses can be a daunting task, further complicated if a university wishes to issue stipends or provide other direct monetary support (covering cost of parking, training materials, food). Additionally, if students interact with patients, electronic medical records or other proprietary information, the University needs to ensure that requirements for background checks, immunization clearance, confidentiality, and minimum mandatory training are met. Policies may exist that allow such students function as “observers” without undergoing the entire onboarding process but also limit these students’ ability to access systems and shadow existing employees. Onboarding students via formal pathways ensures compliance with local policies and procedures, and, potentially, opens the door for remuneration. The timing of an onboarding process, which sometimes can be rather lengthy, needs to be taken into consideration if the program must meet grant deadlines or other sponsor expectations. Issuance of university email addresses, badges, and campus accounts enables use of most systems, and should be limited for the duration of training. Program owners are responsible for completing University-based onboarding processes as well as program-specific onboarding (Supplemental Materials: Sample Onboarding Plan). This may include access to learning management systems, completing various checklists (i.e., goals, expectations, learning plan, contacts, and comportment), adding students to scheduling, etc. If a student does not have a personal laptop that meets University standards or internet access at home, a partner CBO may be able to assist. While some programs accept on a rolling basis, a more typical structure is cohorts that are accrued once or twice a year. A typical cohort size is in single digit numbers due to significant experiential components of these programs (Supplemental Materials: tabular compilation of strategies). State-registered apprenticeships represent a special case among pathway programs. These are structured training programs combining classroom learning with paid work experience that are formally recognized by a U.S. Department of Labor-approved State Apprenticeship Agency. Programs must meet federal quality, safety, and wage standards, and standardized requirements for training and work exposure. Only two AMCs have established apprenticeship programs (see below).

#### Exemplar. Registered CRP apprenticeships at Duke and Washington University School of Medicine

*Duke launched North Carolina’s first state-registered Clinical Research Apprenticeship Program in partnership with Durham Tech and Apprenticeship NC (Strategy AO5, Supplemental Materials). The apprenticeship provides paid work-based learning for individuals affiliated with Durham Tech’s Clinical Trials Research Associate degree program. Apprentices will benefit from competency-aligned mentorship and direct pathways into full-time CRP roles at Duke upon successful completion.*

*Duke University closely collaborated with Washington University School of Medicine (WashU), which previously established t*he Clinical Research Professional *Apprenticeship with the U.S. Department of Labor (DOL). The DOL Clinical Research Professional Apprenticeship is a competency-based, 12-month program that combines 240 hours of structured classroom learning and 2000 hours of hands-on experience.*

*Approved in 2023, the program was officially registered with the DOL as part of the National Apprenticeship System. It serves to attract talent from the region and creates career pathways for individuals who might not have met traditional qualifications for entry into clinical research roles. The first cohort of four has completed the training.*

### Training curriculum, training staff, SMEs, and mentors

In person training is the predominant modality for these programs. Most training programs are developed in-house, primarily based upon the JTF Competency Domains with additional local content [7]. A gap analysis is recommended to determine the program content vis-a-vis CRC competencies. A dedicated program manager provides administrative oversight ensuring consistency of training and enabling a single point of contact. However, most programs also attract additional SMEs for specific topics such as Compliance and IRB submissions. Structured training may be incorporated alongside with on-the-job experience or presented as a separate step in the program. Structures vary from daily to a few times a week, depending on the organizational environment, funding, partnerships, student population, and a host of other factors. Most commonly, a training program would be administered a few times per week for 12–16 weeks (about 200 hours of learning). Students especially value hands-on activities, such as mock IRB panel reviews, simulated consent process or simulated research visits (Supplemental Materials: Correlation of typical training curriculum topics with On- the-Job Experience tasks). Completion of standard required training such as CITI or Dangerous Goods Shipping is advantageous for student’s future employment. Considering that most students would not have prior knowledge of medicine, medical terminology courses, and overview of disease pathways may be required. Study halls could be an important addition to the curriculum. Many students feel overwhelmed with the amount of new information, abbreviations, technical terms and simply with the new environment, people and expectations. Adding an additional mentor, independent of the trainer, is used as a strategy to improve student comfort and sense of belonging (Supplemental Materials: tabular compilation of strategies). More often than not, students require financial support during the training period (see Funding below), unless they are enrolled in an academic program.

#### Exemplar. UCSD research visit simulation activity

*The UCSD Clinical Research Training Program emphasizes simulation-based training in its curriculum (Strategy TC5, Supplemental Materials). The simulation of a research visit included two days of hands-on teaching. On day one, students engaged in pre-visit planning and site visit readiness logistics. On day two students participated in a simulated research visit. Each student was paired with a teacher-actor portraying a mock participant. The actors received detailed descriptions including medical history and medication information and were instructed to simulate realistic behaviors during the visit, including variable levels of engagement, recall, and cooperation. Instructors and support teaching staff assumed various roles encountered in clinical environment. The students conducted and documented typical research visit activities. Individualized feedback was provided to students, and many reported increased confidence and preparedness for real-world clinical research roles.*

### Shadowing and internship opportunities

Shadowing a clinical research coordinator while in the program is fundamentally different from the true on-the-job experience. During shadowing a student has limited opportunities to interact with study participants, access informational systems or prepare documents. Shadowing is meant to be a process of observation and can be accomplished under university policies that allow community presence on campus. This discrete experience provides a closer look at the CRC career path and can be completed in a few hours or over several days. It is important to ensure that the hosting teams are aware of what tasks, if any, the student may be able to do under the shadowing arrangement (if accomplishing specific tasks is desired). On the other hand, on-the-job experience (on-the-job training, internship or apprenticeship, summarily designated as OJE) allows for performing clinical research tasks under direct supervision as a member of a clinical research team. Depending on the administrative onboarding strategy (see above), the OJE is typically full-time and could last from several months to several years as a paid position. Job descriptions for OJE vary widely. A useful resource for establishing on-the-job tasks is O*NET database [1]. Occupation-Specific Information for Clinical Research Coordinator (code 11-9,121.01) lists 33 tasks pertinent for this profession. If the program is registered as a state apprenticeship, an apprentice has to experience at least 50% of the listed tasks. To note, O*Net indicates specific vocational preparation for CRCs of over 2 years and up to 10 years. This is defined as the amount of time “required by a typical worker to learn the techniques, acquire the information, and develop the facility needed for average performance in a specific job-worker situation.” The on-the-job experience cohorts are typically small, ranging from 2 to 5 individuals. Mentors play a large role in on-the-job experience and may receive an allowance (typically ranging from $500-$1000) for their expanded responsibilities. Most programs do not guarantee jobs post OJE completion. However, support with preparation of resumes and job interviews is commonly offered. Participation in on-the-job experience also offers an exposure to hiring managers, improving chances of obtaining a career position after program completion (Supplemental Materials: tabular compilation of strategies).

#### Exemplar: JHU and MUSC mentor trainees via a shared CRP pool

*Programs at the Medical University of South Carolina (MUSC) and Johns Hopkins University (JHU) integrate trainees within an existing fee-for-service float pool through full-time, benefit-eligible, paid positions. Trainees are mentored by existing team members. Researchers can access experienced coordinators on an hourly basis for partial or full study support, enabling flexibility and efficiency. After completing 200 hours of didactic coursework, trainees shadow their peers within the float pool and gradually assume increasing responsibility with mentor support. Many MUSC graduates remain with the float pool and have grown into mentors and managers. At JHU, over 50% of trainees are retained at the university in high-level positions, and overall, 90% are in various research and/or healthcare positions within industry, foundations and other employers.*

### Program evaluation

Evaluations are essential for both training and on-the-job components. Commonly, pre and post-tests are used to assess knowledge acquisition during the training period. Evaluations are either created in-house or may utilize third-party vendors. However, since knowledge acquisition does not necessarily translate into skill, the evaluations during the on-the-job experience are critical. Common methods of evaluating the application of competencies and skills aligned with the JTF Competency Domains include real-time feedback from mentors/ preceptors during the on-the-job experiences. Mentors could provide evaluation in a variety of modalities, both informal and formal (verbal, checklist, critique of demonstrated application). Self-assessment is another mechanism to demonstrate translation of skills into practice. Such self-perceived competencies should be combined with the independent assessment by the mentor. Scoring rubrics, such as Likert scale, provide consistency and uniformity in evaluation of competences and skills. Systematizing evaluation principles among CTSAs with such training programs could be invaluable for establishing baseline quality and effectiveness (Supplemental Materials: tabular compilation of strategies).

#### Exemplar. VCU case study: Leveraging an established evaluation tool

*The CRC Intern Program administered through the CTSA Wright Center evaluates intern progress utilizing mentor feedback and assessment (Strategy EA4, Supplemental Materials) together with the validated Competency Index for Clinical Research Professionals (CICRP-II) self-assessment tool (Strategy EA1, Supplemental Materials) [8]. This 20-item, Likert scale (“not at all confident” to “completely confident”) measures CRP core competencies and is administered pre- and post-internship. Among 24 interns, the median total CICRP-II score increased by 85.6% following internship completion. While pre-internship scores suggest overconfidence from prior experiences, the CICRP-II results demonstrate success in interns’ preparedness to function in a CRC role. Additionally, evaluation data shows a strong correlation between the internship and a continued interest in clinical research careers.*

### Funding

Implementation of a pathway program requires significant funding. Specifically, financial support for students from socio-economically disadvantaged populations allows a reduction in current workload to focus on successful program completion. However, lack of funding continues to be one of the largest barriers to program inception. Until recently, the authors have not seen Federal grants focused on fostering CRP workforce development. Funding for existing programs often comes from institutional funds, donations, and City/State workforce funds. Even with funding, providing stipends, reimbursement or incentives to trainees may be administratively challenging. If the trainees are not AMC hires, renumeration can be administered via a CBO or a staffing contractor (Supplemental Materials: tabular compilation of strategies).

#### Exemplar: Rutgers Health leverages workforce development seed funds

*The entry-level clinical research workforce development initiative at Rutgers Health sits within the Master of Science, Clinical Research Management (MS CRM) program at the School of Health Professions. As part of the initial NCATS award, NJ ACTS earmarked funds for workforce development (Strategy SF1, Supplemental Materials). The goal of the seed funds was to accelerate education and training to fill system-wide clinical research coordinator vacancies. Students in the Academic Clinical Research Management track with academic excellence (GPA 3.3 and above) received one-time program specific academic scholarship funds. The program is now in its 4* ^***th***^ *cohort and shows signs of meeting the identified goal, as 100% of students are placed within months of their graduation.*

## Results

Table 1 summarizes the results of the author’s CRP pathway programs and is not fully representative of all similar programs. Most programs were initiated within the last decade in response to CRP workforce shortages. Notably, most programs admit relatively low number of students per cohort or time period. The low numbers are reflective of considerable complexity of the process, including all barriers discussed above, as well as right sizing the cohorts to hiring demand. Trainees also pose considerable strain on the research teams during shadowing and OJE portions of the program. Nevertheless, low cohort numbers enable high touch in instilling necessary CRP competencies, resulting in high completion and job entry rates. Most graduates remained at the universities, but not all ended with employment as CRC. Some continued their academic studies or obtained positions in other healthcare-related fields. The graduates not only infuse economy with salaries, but also convey secondary benefits, such as increased awareness of research as a profession, better understanding of the research process, and improved impression of universities as community partners.

**Table 1.** Summary of the internship/apprenticeship programs of the author CTSAs, in an alphabetical order Table 1 long description.

Location	Years of the program	Students enrolled	Graduated/ completed the program	Obtained positions in research and other healthcare-related position	Comments
Duke University (internship)	4	47	47	25	In addition, 7 interns continued their education, and 15 are unknown or not captured
Johns Hopkins University	12	61	56	56	Apprentices are hired to full-time, benefit-eligible positions. Graduation is defined as those who were promoted from Apprentice to a higher Research position or chose to pursue higher education in a healthcare field.
Medical University of South Carolina	12	14	12	12	All graduates remained in the research fee-for-service float pool and were promoted to the next CRC level after graduation
Rutgers Health	4	6	6	6	Pilot and first cohort graduated with job placements in clinical research, two additional cohorts in progress (*N* = 14)
University of California, San Diego	1	4	4	2	The program completed one cohort and will be resuming after being on hold due to funding challenges.
University of California, Davis	4	33	32	22	In addition to community students, the program is also leveraged for existing employees cross-training, with over 30 UC Davis employees graduating from the program.
University of Minnesota, Rochester	2	3	2	2	As of the time of writing of this manuscript, an additional 7 students are in the process of completion of their internship
Virginia Commonwealth University	4	38	38	7	The program focuses on academic credit and fulfills the internship requirements of an undergraduate academic health-related course.
Washington University School of Medicine (Apprenticeship)	2	4	4	4	Upon program completion all apprentices were offered a CRC position

## Conclusions

CRP pathway programs are critically important in meeting and sustaining staffing needs at AMCs and beyond. The landscape survey conducted by ACTS Strategic Interest Group (2025 poster) unveiled multiple models in use. This manuscript builds on these findings by further detailing and summarizing the innovative pathway models that improve our ability to expand awareness of CRP roles and create training opportunities. While the described programs do not expressly focus on historically underrepresented applicants, lessons learned could be applied with this focus in mind. Similarly, the authors acknowledge the need to adapt and expand CRP-focused entry programs for other research-related professions, such as regulatory, data, quality assurance, and others. Additional data gathering of pathway models into these professions is necessary.

In September 2025, ACRP received a two-year, $50,000 conference grant (R13) from NIH NCATS to advance their mission to ensure a representative, research-ready, and sustainable clinical research workforce essential to advancing therapies that improve global human health. The grant (Award Number: 1R13TR005703-01) will fund a two-part annual conference session series, “Advancing Clinical and Translational Science through Building a Research-Ready Clinical Research Professional Workforce in the U.S.,” and will bring together a wide array of stakeholders specifically to discuss innovative models for career entry and retention [9]. The authors believe that this conference represents a significant milestone in solidifying our gains in CRP workforce development.

## Supporting information

10.1017/cts.2026.10806.sm001 Marusina et al. supplementary material Marusina et al. supplementary material

## Supplementary material

The supplementary material for this article can be found at https://doi.org/10.1017/cts.2026.10806.

## Table 1. Long description

The table presents data on internship and apprenticeship programs at various universities. It includes information on the years of the program, number of students enrolled, number of graduates, positions obtained in research and other healthcare-related fields, and additional comments. The table has 10 rows and 6 columns. The columns are labeled as Location, Years of the program, Students enrolled, Graduated/completed the program, Obtained positions in research and other healthcare-related positions, and Comments. The rows list different universities and their respective program details. For example, Duke University (internship) has been running for 4 years with 47 students enrolled and 47 graduates, of whom 25 obtained positions in research and other healthcare-related fields. Johns Hopkins University has a 12-year program with 61 students enrolled and 56 graduates, all of whom obtained positions. The Medical University of South Carolina also has a 12-year program with 14 students enrolled and 12 graduates, all of whom obtained positions. Rutgers Health has a 4-year program with 6 students enrolled and 6 graduates, all of whom obtained positions. The University of California, San Diego has a 1-year program with 4 students enrolled and 4 graduates, of whom 2 obtained positions. The University of California, Davis has a 4-year program with 33 students enrolled and 32 graduates, of whom 22 obtained positions. The University of Minnesota, Rochester has a 2-year program with 3 students enrolled and 2 graduates, both of whom obtained positions. Virginia Commonwealth University has a 4-year program with 38 students enrolled and 38 graduates, of whom 7 obtained positions. The Washington University School of Medicine (Apprenticeship) has a 2-year program with 4 students enrolled and 4 graduates, all of whom obtained positions. The comments section provides additional context for each program, such as the continuation of education for some interns, the promotion of apprentices to full-time positions, and the focus on academic credit for some programs.

Navigate back to Table 1..

## References

[1] O*Net On Line, sponsored by the U.S. Department of Labor. Occupational profile, clinical research coordinators. (https://www.onetonline.org/link/summary/11-9121.01) Accessed September 18, 2025.

[2] Association for Clinical Research Professionals (ACRP). Addressing clinical research workforce crisis: a call for collective action. (https://acrpnet.org/insights/white-papers/addressing-the-clinical-research-workforce-crisis-a-call-for-collective-action) Accessed September 18, 2025.

[3] Perez-Johnson I , Holzer H. The importance of Workforce Development for a Future: Ready, Resilient, and Equitable American Economy (WDEMP-importance-of-workforce-development-brief-April-2021.pdf). American Institutes for Research (AIR). 2021. Accessed September 18, 2025.

[4] Sonstein SA , Jones CT. Joint task force for clinical trial competency and clinical research professional workforce development. Front Pharmacol. 2018;9:1148. doi: 10.3389/fphar.2018.01148. PMC6198073 30386238

[5] Stevens E , Watkins-Keller G , Reilly N , Panettieri RA , Tafuto B. Real-world mastery of essential skills in a clinical research coordinator graduate program. J Clin Transl Sci. 2025;9:e162. doi: 10.1017/cts.2025.10077. PMC12392342 40895452

[6] Traynor SM , Locklear TD , Freel SA , Batch BC , Davis LP , Faulk TC , Freeman D , Cranfill JR , McMillan AE , Budinger S , Snyder DC , et al. Building pathways to clinical research careers: partnerships with academic programs to provide students with experiential learning opportunities in clinical research. Acad Med. 2025;100:1272–1277. doi: 10.1097/ACM.0000000000006176. PMC13334718 40720785

[7] Marusina K , Kishchenko O , Griffiths A. Join the team” clinical research workforce development model: a new paradigm in healthcare career opportunities. J Clin Transl Sci. 2024;8:e128. doi: 10.1017/cts.2024.577. PMC11428055 39345706

[8] Hornung CA , Ianni PA , Jones CT , Samuels EM , Ellingrod VL. Indices of clinical research coordinators’ competence. J Clin Transl Sci. 2019;3:75–81. doi: 10.1017/cts.2019.381. PMC6759060 31552144

[9] Association for Clinical Research Professionals (ACRP). ACRP receives NIH grant to strengthen U.S. Clinical Research Workforce. (https://acrpnet.org/2025/09/03/acrp-receives-nih-grant-to-strengthen-u-s-clinical-research-workforce) Accessed September 18, 2025.

