# Supplementary material for: Reimagining entryways: How work-based learning at Clinical and Translational Science Award (CTSA) centers is expanding access for the next generation of clinical research professionals
Source: J Clin Transl Sci. 2026 Jul 27;10(1):e142. doi: 10.1017/cts.2026.10806 (PMC13748993; doi:10.1017/cts.2026.10806)
Supplement: Supplementary file 1 [file S2059866126108061sup001.docx]

**Supplemental Materials: Sample Onboarding Plan**

1. **Administrative processes**
   a. Obtaining university email
   b. Access to systems
   c. Badges
   d. Parking
   e. Timecards/attendance process
   f. Location-specific requirements such as background checks/ vaccinations/ testing
2. **Required Compliance Training (examples)**
   i. Annual Compliance training
   ii. Annual HIPAA, Privacy and Security
   iii. Incident Reporting System
   iv. Any other training, such as Cybersecurity, Violence Prevention in Workplace, Disaster Preparedness and others as required)
   v. Environmental Health and Safety
   vi. University policies and procedures relevant to research
   vii. Clinical setting related training
3. **Clinical Research training** (see Supplemental material –Training)
4. **Unit (Department/Division/Center) training**
5. **Listings of additional resources (examples)**
   a. Research Staff listserv and Newsletters
   b. LinkedIn communities
   c. Instructor-Led Courses
   d. Professional organizations and networks
   e. Research vaults (compendiums of research-related processes, procedures, workflows)

**Supplemental Materials: Correlation between typical training curriculum topics (not exhaustive) and on the job experience**

| **Competency Domain** | **Potential Training Curriculum topics** | **Sample of On the Job Experience Activities** |
| --- | --- | --- |
| **Scientific Concepts and Research Design** | CITI modules as required  Medical Terminology  Health literacy  Introduction to clinical trials  Research Administration  Study Design, Data, Ethics, FDA Regulation, & Team Science  Biostatistics and research Design |  |
| **Leadership and Professionalism** | Resume and interview preparation  Professionalism in Workplace  Optimizing communication with stakeholders | Learn about communication with stakeholders, including study sponsors, investigators, study staff, ancillary services. Help to schedule and coordinate site visits or to answer questions about issues such as incomplete data. |
| **Ethical and Participant Safety Considerations** | Informed Consent  Responsible Conduct of Research | Eligibility and Consent: Learn to Assess eligibility of potential subjects through methods such as screening interviews, reviews of medical records, or discussions with physicians and nurses. Observe subject enrollment to learn how informed consent is properly obtained and documented. Under direct supervision inform patients or caregivers about study aspects and outcomes to be expected. Track enrollment status of subjects and document dropout information, causes and retention efforts. Understand how to use Electronic medical records and appropriately manage PHI. Understand a concept of vulnerable populations |
| **Investigational product development** | Investigational Product management  Safety management and reporting  Good Manufacturing Practices | Learn about handling of Investigational Drugs: understand the process for ordering drugs or devices necessary for study completion. Under direct supervision discuss use of medical devices/drugs with patients. Understand dosages (single dose, maximum tolerated dose) and route of administration |
| **Clinical Study Operations** | IRB submissions and reporting  Clinical Trials Oversight and Delegation  Protocol and GCP Compliance Essential Trial Documents  Source Documentation  Participant recruitment  EMR Research Functionalities  Blood-borne pathogens and Dangerous Goods Shipping  Biospecimen Collection and Processing  Running a research visit  Good Laboratory Practices  Good Export Controls  Readability Fundamentals for Clinical Research Participant Engagement Materials  Disease-specific training (i.e. cancer, stem cell, immunology) | Basics of coordination: learn how to schedule subjects for appointments, procedures, or inpatient stays as required by study protocols. Contact outside health care providers and communicate with subjects to obtain follow-up information.  Learn how to prepare and maintain study-related documentation, such as protocol worksheets, source documents, procedural manuals, delegation logs, consents, adverse event reports, or progress reports, case report forms (CRF), drug dispensation records, or regulatory forms (IRB documents).  Learn how to perform specific protocol procedures such as interviewing subjects, administering questionnaires, taking vital signs, and performing electrocardiograms, and others.  Observe/participate in sample collections: under direct supervision participate in the requisition, collection, labeling, storage, or shipment of specimens. Communicate with laboratories or investigators regarding laboratory findings  Learn to identify protocol problems (deviations), understand problem resolution methodologies, such as protocol revisions or corrective and preventative action plans.  Participate in Recruitment: Confer with health care professionals and study team members to determine the best recruitment practices for studies. Understand the development of advertising and other informational materials to be used in subject recruitment.  Learn to record adverse event and side effect data, and reporting of events to oversight agencies.  Learn clinical research systems in sandbox environment |
| **Study and Site Management** | Study feasibility and start up  Budgets and invoicing  Research Finance  Quality Assurance in research | Learn basics of QA and Monitoring: participate in/observe quality assurance audits conducted by study sponsors, federal agencies, or specially designated review groups.  Observe/assist on monitoring visits to ensure compliance with protocols and with all relevant local, federal, and state regulatory and institutional polices. Understand purpose and use of regulatory binders  Understand principles of preparation and management of research budgets, activities in EMR leading to billing and invoicing. Observe/participate in monetary disbursements (participant stipends)  Understand how Study Feasibility is conducted: participate in evaluation of proposed study protocols; learn about factors that may influence feasibility, such as procedures, resource availability, sample collection processes, data entry process and volume  Learn how to organize Space: Organize space for study equipment and supplies, understand principles of stewardship of resources |
| **Data Management and Informatics** | Data management; query resolution | Learn principles of Data management: how to enter study data, understand how CRFs work, how to manage query resolution, understand principles of data analysis and evaluation  Experience with REDCap in the sandbox environment |

**Supplemental Materials: A sample Evaluation Rubric**

| Domain Criteria | Range 1-3  Unacceptable | Range 4-7  Needs Improvement | Range 8-10  Range between Meets (8 pts) to Exemplary (10 pts) | Points  Earned |
| --- | --- | --- | --- | --- |
| Timeliness | Missed 20% or more of meetings; significant delay in meeting deadlines | Missed /late for 10% of meetings; missed deadlines by few days to one week | On time for all meetings with advisor/preceptor  All deadlines met based on project plan |  |
| Manages Communication & Contacts | Ignores communication from Preceptor/Advisor | Responds to queries from Preceptor/Advisor by one week | Exhibits timely response to all emails and communication from preceptor; contacts advisor to inform of any unforeseen illness or anticipated tardiness |  |
| Respect & collegiality | Ignores suggestions made by Preceptor/Advisor | Somewhat receptive to suggestions made by Preceptor/Advisor but with push back; adjusts deliverables superficially | Receptive to suggestions & feedback and adjusts deliverables accordingly, or if not, discusses differences in a collegial manner |  |
| Self-Initiative | Rarely takes initiative | Occasionally takes initiative in planning & carrying out the project | Almost always takes initiative in planning & carrying out the project |  |
| Strives for a high level product | Accepts mediocre work | Makes an effort to produce above average work | Strives for Excellence |  |
| Total |  |  |  |  |

Adapted from Rubric for Assessing Professional Development www.uvm.edu/~socwork/msw/pdfs/**Rubric**.pdf‎

**Supplemental Materials: tabular compilation of strategies for each component of a workforce development program**

**Strategies for Community Engagement (CE)**

CE.1 Partner with CTSA Community Engagement/Workforce Development teams

CE2. Develop internet presence via a dedicated website or social media

CE3. Get involved in local chapters of ACRP/SOCRA

CE4. Engage with local biotech organizations

CE5. Present at a Health Occupations Students of America (HOSA) meeting

CE6. Attend local educational events/career fairs, etc.

CE7. Engage with faculty at Community Colleges, especially in health-

related concentrations

CE8. Establish relationships with local CBOs

CE9. Connect with University’s Government and Community Relations

CE10. Connect with the local government Workforce Development Offices

CE11. Collaborate with existing undergraduate and graduate programs

CE12. Attend local job fairs and high school career events

**Communication Strategies with Program Collaborators (PC)**

PC.1 Identify key players and establish a single point of contact for each collaborating unit

PC.2 Define roles and responsibilities, even if informally

PC3. Define and agree upon communication pathways

PC4. Schedule regular check-ins and updates

PC5. Schedule in person milestone meetings, if possible (I.e. kick-off, postmortem)

PC6. Share successes and promote partnership (newsletters, LinkedIn, etc.)

PC7. Consider including key personnel as authors on the relevant manuscripts

PC8. Establish feedback sessions

**Options for Administrative Onboarding (AO)**

AO1. Utilize provisions in the existing policies for community presence on campus

AO2. Onboard via Volunteer Services

AO3. Employment through Temporary Services or a staffing agency

AO4. Employment as an entry level CRP, or other via Human Resources

AO5. State-registered apprenticeship program

AO6. Contingent worker status (non-permanent employee status)

**Tools/formats for Onboarding (TO)**

TO1. Listing of expectations and requirements

TO2. In-person orientation sessions

TO3. Checklist with essential information (see Supplemental Materials)

TO4. “Getting Started” online training

TO5. Learning Plan, including expectations for classroom, conduct, clothing, homework etc.

**Ideas for sourcing Training Content (TC)**

TC1. Purchase commercial vendor materials

TC2. Leverage existing institutional training curriculum

TC3. Collaborate with other CTSAs for shared materials (e.g. DIAMOND Portal)

TC4. Partner with Subject Matter Experts for additional contentTC5.

TC5. Include simulations/practices, such as

- Simulating Informed Consent discussions
- Simulating a Research Visit
- Preparing sample kits
- Accessing EMR Training environment
- Attending mock IRB panel
- Preparing mock source documents
- Creating Regulatory binders
- Completing mock essential documents (1572, Delegation log etc)
- Practicing software workflows in sandbox environments

TC6. Include skills-based learning: phlebotomy, EKG, vitals, blood smears, point-of-care testing

TC7. Include knowledge assessment/evaluations

Sample Shadowing Activities (SA)

SA1. Participate in team meetings

SA2. Attend Sponsor visits

SA3. Observe mentor leading Informed Consent Discussion

SA4. Attend participant study visit (as applicable)

SA5. Learn data collection and entry (may use mock forms)

SA6. Assemble sample kits under observation

SA7. Sit on pre-screening calls with potential research participants

SA8. Engage in participant scheduling activities

SA9. Collect and process biospecimens (as applicable)

SA10. Shadow research ancillary offices (such as IRB, Research Administration, Scientific Review Board, Investigational Drug Pharmacy etc.)

SA11. Shadow research clinic staff (nurses, phlebotomists etc.)

Best Practices for Empowering on-the-job Experience Mentors

- EM1. Provide lists of tasks that an intern needs to complete
- EM2. Ensure clear understanding what an intern can and cannot do
- EM3. Encourage exposure to diverse experiences
- EM4. Provide cultural sensitivity tips and training
- EM5. Provide stipends for mentors
- EM6. Establish frequent communication with the program management

Approaches to Evaluation of pathway programs (EA)

- EA1. Use a validated evaluation tool (e.g. CCRPII)
- EA2. Purchase existing vendor assessment (i.e. ACRP)
- EA3. Develop site specific self-assessment tool based on JTF Competency Domains
- EA4. Include mentor/preceptor observed skills/competencies assessments

**Typical Expense Categories for a pathway program**

EC1. Hourly pay/stipend

EC2. Trainer and program manager salary

EC3. Third party training materials (license/purchase)

EC4. Printed materials

EC5. Parking

EC6. Food during the program

EC7. Technology (computers, company-issued phones, access fees)

EC8. Onboarding fees (i.e. Volunteer Services Fees)

**Possible Sources of Funding**

SF1. CTSA or institutional support

SF2. Philanthropy (i.e. individual donors or philanthropic organizations)

SF3. Foundations or industry grants

SF4. City and State grants for workforce development

SF5. State Apprenticeship funding

SF6. Departmental recharge

SF7. Department funding
